# Isoliensinine: A Natural Compound with “Drug-Like” Potential

**DOI:** 10.3389/fphar.2021.630385

**Published:** 2021-04-22

**Authors:** Yan Cheng, Hong-Li Li, Zi-Wei Zhou, Hui-Zhi Long, Hong-Yu Luo, Dan-Dan Wen, Lin Cheng, Li-Chen Gao

**Affiliations:** ^1^ Department of Pharmacy, Phase I Clinical, Trial Centre, Changsha Central Hospital Affiliated to University of South China, School of Pharmacy, University of South China, Changsha, China; ^2^ Hunan Provincial Key Laboratory of Tumor Microenvironment Responsive Drug Research, Hengyang, China; ^3^ State Key Laboratory of Ophthalmology, Zhongshan Ophthalmic Center, Sun Yat-sen University, Guangzhou, China

**Keywords:** pharmacology, pharmacokinetics, synthesis, mechanism, isoliensinine

## Abstract

Isoliensinine, a bisbenzylisoquinoline alkaloid isolated from *Nelumbo nucifera* Gaertn, exerts a variety of beneficial effects, such as antitumor, cardioprotective, antioxidant, antidepressant, and anti-HIV effects, and ameliorates T2DM with hyperlipidemia and Alzheimer’s disease. In this article, the recent literature on isoliensinine, including its pharmacology, pharmacokinetics, and synthesis and extraction, is summarized. Moreover, possible future prospects and research directions are also discussed. Studies on isoliensinine were found by searching a combination of keywords including “pharmacology,” “pharmacokinetics,” and “synthesis and extraction” in the main databases, including PubMed, Google Scholar, Web of Science, NCBI, and Wan Fang. Many studies have pointed out that a major limitation of isoliensinine is its poor solubility in aqueous media. Considering its advantages and limitations, isoliensinine can be used as a lead compound to develop novel efficient and low-toxicity derivatives. The available literature indicates that isoliensinine displays “drug-like” potential. Additionally, there are many related issues and novel mechanisms that need to be explored.

## Introduction

Natural products have been an important source of new drugs (Katz and Baltz, 2016; Newman and Cragg, 2016). Phytomedicine has played a unique role in the clinical therapy of various diseases for thousands of years (Jaiswal et al., 2016; Mazumder et al., 2018). Paclitaxel is used to treat metastatic breast cancer that has failed to respond to combined chemotherapy (Abu Samaan et al., 2019). Berberine is often used to treat infections, such as gastroenteritis and bacillary dysentery, caused by sensitive pathogens such as *Shigella* and *Vibrio cholerae* (Jin et al., 2016). Chloroquine is of great benefit in treating malaria (Ashley et al., 2018). However, the transition of a natural compound from a “screening hit” to a “drug lead” and then to a “marketed drug” is associated with increasingly challenging milestones.

*Nelumbo nucifera* Gaertn, also known as lotus, is a sacred symbol in religious culture and a dual-purpose plant with edible and medicinal properties (Chen et al., 2019). All parts of lotus are of great commercial value. “Lian Zi Xin,” also named *Nelumbinis Plumula* (the green embryo of the mature lotus seed), is a traditional Chinese medicine that has been used to cure several diseases (Mukherjee et al., 2009; Do et al., 2013; Paudel and Panth, 2015; Sharma et al., 2017; Lin et al., 2019). The Chinese Pharmacopoeia indicates that isoliensinine possesses the functions of clearing the heart and calming the nerves, communicating with the heart and kidneys, and promoting astringent essence and hemostasis. As a result, isoliensinine is mainly used to remedy symptoms such as the invasion of heat into the pericardium, dizziness and delirium, incompatibility between the heart and kidney, insomnia, spermatorrhea, blood heat, and vomiting (Commission, 2015).

Isoliensinine (IUPAC name (1*R*)-1-[[4-hydroxy-3-[[(1*R*)-6-methoxy-1-[(4-methoxyphenyl)methyl]-2-methyl-3,4-dihydro-1H-isoquinolin-7-yl]oxy]phenyl]methyl]-6-methoxy-2-methyl-3,4-dihydro-1H-isoquinolin-7-ol) is a phenolic bisbenzylisoquinoline alkaloid that was first isolated and named “Lien Tze Hsin” by the Japanese scholar Ma-sao from Chinese Formosa (Tomita et al., 1965). Later studies showed that all parts of the lotus, such as the rhizome, leaf, and seed, could be used to obtain isoliensinine, but the contents of various alkaloids obtained from the same part of the plant differs depending on the location of origin and method used to dissect the lotus (Zhao et al., 2014).

In the past 2 decades, there have been many reports on the pharmacology, mechanisms of action, and effects of isoliensinine, including its antitumor activity, cardiovascular protection, improvement of symptoms of diabetes and Alzheimer’s disease, anti-HIV effects, and reversal of bleomycin-induced pulmonary fibrosis. Additionally, isoliensinine was predicted to have great medicinal potential by the online scoring tool Swiss ADME (http://www.swissadme.ch/index.php), which assesses “drug-likeness” through a series of indexes, such as the Lipinski, Ghose, Veber, Egan, Muegge, and Bioavailability Score indexes (Daina et al., 2017). In this article, the pharmacological action and associated molecular mechanism, pharmacokinetic characteristics, chemosynthesis, and biosynthesis of isoliensinine were summarized predominantly by researching major online databases. To the best of our knowledge, this is the first report to independently review the pharmacological properties of isoliensinine to understand its drug potential. The review also critically describes the limitations of isoliensinine.

## Isolation, Purification, and Identification

In 1962, Chao et al. (Chao et al., 1962) reported the isolation of liensinine from the traditional Chinese medicine “Lien Tze Hsin,” the seed embryo of lotus. Isoliensinine was first extracted from “Lien Tze Hsin” in 1965 by Tomita et al. (Tomita et al., 1965). The source of isoliensinine is shown in Supplementary Figure S1. It is difficult to isolate and purify isoliensinine, liensinine, and other alkaloids extracted from the green embryo of lotus due to their similar physicochemical properties and structures. Therefore, several advanced methods of extraction and purification have been established. Preparative countercurrent chromatography (CCC) isolation of isoliensinine and its analogs was successfully performed for the first time from the embryo of the seed of *Nelumbo nucifera* Gaertn using an upright coil planet centrifuge by Wu et al. (Wu et al., 2004). Recently, many effective methods with high purity and low reaction time have been established by changing the two-phase solvent system of CCC, such as replacing the mobile phase of HCl as a pH regulator with a safer Na_2_HPO_4_/NaH_2_PO_4_ buffer solution and lysine. (Duanmu et al., 2010; Wang et al., 2010; Wang et al., 2017). In addition, Fang et al. (Fang et al., 2017) utilized an ionic liquid as a pH zone–refining reagent for the separation of alkaloids from *Nelumbo nucifera* Gaertn, and they succeeded in isolating six alkaloids, including *N*-nornuciferine, liensinine, nuciferine, isoliensinine, roemerine, and neferine. In addition, liquid chromatography coupled to diode array detection and tandem mass spectrometry has also been established for the separation, identification, and rapid determination of isoliensinine by Chen et al. (Chen et al., 2007). Isoliensinine in lotus leaves was separated with nonaqueous capillary electrophoresis coupled to ultraviolet and mass spectrometry in 2013 (Do et al., 2013). Most recently, to avoid the interference of solvent peaks in the determination of capillary electrophoresis, liensinine, isoliensinine, rutin, and hyperoside were successfully separated from *Nelumbinis plumula* by far infrared–assisted removal of extraction solvent (Wan et al., 2019). In general, the methods used to separate, purify, and identify isoliensinine are becoming more convenient, safer, and more effective.

## Chemosynthesis and Biosynthesis

In 1969, Karnetani et al. reported the chemical synthesis route of isoliensinine (Kametani et al., 1969; Supplementary Figure S2A). Cyclization of amide 3) obtained by condensation of 4-benzyloxy-3-methoxyphenethylamine 1) and 4-methoxyphenylacetic acid 2) at 180–190°C underwent a Bischler–Napieralski reaction to yield 3,4-dihydroisoquinoline (4), which further gave rise to 4′-*O*-methyl-*N*-methylcoclaurine 7) through multiple reactions (5, 6). Next, the Ullmann reaction was carried out between 4′-*O*-methyl-*N*-methylcoclaurine 7) and 4′,7-*OO*-dibenzyl-3′-bromo-*N*-methylcoclaurine 8) and produced diastereoisomeric racemates of *OO*-dibenzylisoliensinine (9). Finally, isoliensinine was obtained *via* hydrolysis of *OO*-dibenzylisoliensinine. In addition to chemical synthesis, isoliensinine can also be obtained *via* biosynthesis (Supplementary Figure S2B). Isoliensinine belongs to the benzylisoquinoline alkaloid (BIA) family. To date, over 2500 BIAs have been discovered, and many of them have been employed in clinical treatment. These compounds have many complex structures (Facchini and De Luca, 2008; Ziegler and Facchini, 2008). Most BIAs follow a common metabolic pathway before forming the last component. The biosynthesis of BIAs initially involved the condensation of two tyrosine derivatives, dopamine produced by L-dopa catalyzed by TYDC and 4-hydroxyphenylacetaldehyde produced by amide deamination. After being catalyzed by NCS, the intermediate (*S*)-norcoclaurine was produced. (*S*)-Norcoclaurine further underwent two reactions catalyzed by 6OMT and CNMT to form (S)-*N*-methylcoclaurine, which was catalyzed by Cyp80A1 to generate BIAs (Glenn et al., 2013; Diamond and Desgagné-Penix, 2016).

## Pharmacokinetics

In the last 20 years, researchers have clarified the *in vivo* metabolic process of isoliensinine through different experimental models, including mice, beagle dogs, and human plasma. Pharmacokinetic studies in rats showed that the oral bioavailability of total bisbenzylisoquinoline alkaloids reached 62.5%, and double peaks were observed on the concentration-time curve of isoliensinine when rats were given a single dose of 20 mg/kg (Huang et al., 2011). Nevertheless, the double peak phenomenon does not exist in other pharmacokinetic reports (Hu et al., 2015; Peng et al., 2015). After intravenous administration, isoliensinine was metabolized *via* a two-compartment open model in rats. Isoliensinine was mainly eliminated, as indicated by t_1/2β_>t_1/2_, and widely distributed in rat tissue, as indicated by Vd (0.647 ± 0.091 L/kg), which is much larger than the volume of rat body fluid (Chen and Li, 2011). Similarly, isoliensinine pharmacokinetic parameters exhibited few differences in different experiments. Moreover, all of the available literature has confirmed that isoliensinine samples are very stable regardless of long-term storage and repeated freezing and thawing (Huang et al., 2011). Based on high-performance liquid chromatography and data-dependent electrospray ionization tandem mass spectrometry, Zhou et al. discovered three new-phase I metabolites in the liver microsomes of beagle dogs, namely, 2-*N*-desmethylisoliensinine, 2′-*N*-desmethylisoliensinine, and 2′-*N*-6-*O*-didesmethylisoliensinine, indicating that isoliensinine was mainly metabolized in the liver through *N*-demethylation and *O*-demethylation (Zhou et al., 2012). It has been reported that neferine is mainly metabolized by CYP450 enzymes, including CYP3A, CYP2B, and CYP2D6, so they also assumed that isoliensinine was metabolized by CYP450 enzyme systems, but the specific enzyme involved in this process was not clear (Huang et al., 2007; Shen et al., 2014).

Furthermore, interactions between isoliensinine and other protein molecules have been investigated. Experiments in Caco-2, MDCK, MDCK-MDR1, and MDCK-MRP2 cells confirmed isoliensinine as a substrate of the efflux transporter P-gp, but MRP2 did not participate in the efflux transport process (Yu et al., 2013). Another study clarified that liensinine and dauricine were substrates of BCRP but tetrandrine, isoliensinine, and neferine were not. Interestingly, the membrane permeability of all five bisbenzylisoquinoline alkaloids is not high (Tian et al., 2013). By establishing an *in vitro* liver microsomal enzyme system, Nan Li et al. confirmed that isoliensinine and CYP3A had a weak interaction that did not affect the metabolism of CYP3A substrates, which indicated that isoliensinine can be used together with CYP3A substrate drugs (Nan and Xuan, 2012). In summary, the evidence clearly indicates that only the ABC transporter P-gp regulates the intracellular concentration of isoliensinine and affects its clinical effects.

## Pharmacology

There have been a large number of pharmacological reports on isoliensinine since it was extracted from “Lian Zi Xin.” In the beginning, the cardioprotective action and related molecular mechanisms were initially investigated. Recently, isoliensinine was demonstrated to possess potent antitumor activity. In addition, isoliensinine, a major active ingredient of the traditional Chinese medicine “Lian Zi Xin,” has displayed a series of beneficial effects on various organs, such as the liver, lung, and kidney. The specific pharmacological activities are shown in Table 1.

**TABLE 1:** Pharmacological activities of isoliensinine.

Pharmacological activity	Model	Action and mechanism	References
Antioxidant activity	Human hepatocellular HepG2	Anti-oxidative stress	Xie et al. (2013)
		ROS↓, TBARS↓, LDH ↓	
		GSH↑	
	d-Galactose–induced aged mice	Antiaging	Liu et al. (2011); Shen et al. (2017)
		MDA↓, SOD↑, GSH-Px↑	
	Rats, primary glomerular cells	Protecting kidney	Deng (2015)
		NADPH↓, p22phox↓, p67phox↓	
Antidepressant activity	Mice (the forced swimming test)	Anti-immobility effects	Sugimoto et al. (2015)
		5-HT _1A_ receptor	
Improving Alzheimer’s disease activity	Cells, PC12; rats	BchE↓, AChE↓, ROS↓, Ca^2+^ channel↓, p-Tau↓, CaM↓	Lin et al. (2013)
Antidiabetic activity	Rat skeletal muscle cells, L6; the KK-Ay rats	GLUT4↑, *p*-AMPK↑, *p*-ACC↑, PPARγ↓, SREBP-1c↓, ACC↓	Yang et al. (2017)
Anti-pulmonary fibrosis	Male Kunming mice	MDA↓, hydroxyproline↓, ALP↓	Xiao et al. (2005); Tang et al. (2007)
		SOD↑, TGF-β1↓, TNF-α↓,MMP2↓	
Dilated bronchus	Isolated mouse airway smooth muscle	Relaxation of the ASM	Liu et al. (2019)
		LVDCC↓	

↓, downregulation, inactivation, inhibition; ↑, upregulation, activation.

### Anticancer Activity

There have been increasing numbers of reports on the antitumor effect of isoliensinine since 2014. Isoliensinine targets different signaling pathways at similar doses to exert antitumor activity. The specific molecular changes are shown in Supplementary Figure S3.

#### Autophagy Enhancement

Law et al. (Law et al., 2014) reported that isoliensinine was a small-molecule autophagy enhancer and induced apoptosis-resistant cancer cell death. Their study revealed that isoliensinine induced autophagy in various cells, including MCF-7, PC-3, Hep3B, A549, H1299, and LO2 cells. However, isoliensinine only selectively led to cancer cell death in an autophagy-mediated manner. Isoliensinine induced autophagy by activating the AMPK–TSC2–mTOR signaling pathway in cervical cancer. The formation of GFP-LC3, a marker of autophagy, significantly declined in HeLa cells pretreated with an AMPK inhibitor. The same result was also observed in Atg−/− mouse embryonic fibroblasts (MEFs) but not in Atg+/+ wild-type MEFs. Moreover, the authors found that isoliensinine markedly induced cell death in several cell types, including apoptosis-defective cells, such as caspase-3/7/8–deficient MEFs, and apoptosis-resistant cells, such as Bax/Bad double knockout MEFs.

#### Cell-Cycle Arrest and Induction of Apoptosis

Compared with liensinine and neferine, isoliensinine had the strongest antitumor effect in TNBCs. Zhang et al. (Zhang et al., 2015) demonstrated that isoliensinine selectively led to cycle arrest at G1 phase and induced apoptosis in TNBCs. On the one hand, isoliensinine at a dose range of 10–40 μM increased the expression of p21, which was accompanied by decreased cyclin E1. On the other hand, isoliensinine provoked apoptosis *via* the mitochondrial pathway, which involved the downregulation of Bcl-2 and the upregulation of Bax, cleaved caspase3, cleaved caspase9, and PARP-1. The authors further investigated and verified that isoliensinine activated the p38MAPK/JNK signaling pathway, and the generation of ROS was increased in TNBCs. Moreover, p38 MAPK activation induced ROS generation but JNK activation did not.

Hepatocellular carcinoma (HCC) is the second leading cause of cancer-related mortality worldwide. However, the choice of treatment drugs for patients, especially advanced patients, is limited (Forner et al., 2018). Fortunately, Shu et al. (Shu et al., 2015; Shu et al., 2016) confirmed that isoliensinine selectively induced HCC apoptosis both *in vitro* and *in vivo*. Their study revealed that isoliensinine inhibited NF-κB activity and the constitutive phosphorylation of p65 in HCCs. Moreover, they observed that isoliensinine (3–10 mg/kg) suppressed tumor growth in Huh-7 xenograft nude mice *via* the same mechanism. In addition, the safety and bioavailability of isoliensinine in Kunming mice transplanted with H22 cells were clarified. Next, they found that isoliensinine directly impaired the PP2A/I2PP2A interaction so that the expression of PP2A was upregulated, which triggered dephosphorylation of P65 at Ser536. In conclusion, isoliensinine is a potential choice for cancer treatment.

#### Synergistic Anticancer Activity

Chemoresistance is becoming an increasingly serious problem for several chemotherapeutic drugs that directly leads to treatment failure, poor prognosis, and cancer recurrence (Zheng, 2017). Isoliensinine has been demonstrated to augment the anticancer effect of cisplatin by enhancing the intracellular uptake of cisplatin and ROS-mediated apoptosis in colorectal cancer cells. Combinatorial regimens of cisplatin with isoliensinine significantly induced cycle arrest at S phase, apoptosis, morphological changes, accumulation of intracellular calcium, dissipation of MMP, and activation of the MAPK/PI3K/AKT pathway compared to individual reagent intervention. Furthermore, combination regimens provoked downregulation of Bcl-2 and upregulation of cytochrome C, cleaved caspase-3/9, and cleaved PARP. Another study also demonstrated the same effect; isoliensinine in combination with dauricine obviously augmented its anticancer activity in HCC cells by suppressing glycosis. Mechanistically, combination intervention with isoliensinine and dauricine dramatically elevated the expression of miR-199a accompanied by substantial downregulation of HK2 and PKM2, which repressed glycolysis and increased the chemosensitization of HCC cells. Thus, combinatorial regimens, such as combining phytochemicals with potent anticancer activity with approved chemical drugs, seem to be a beneficial strategy for chemoresistant conditions (Li et al., 2018; Manogaran et al., 2019).

### Cardiovascular Protective Effect

Under the guidance of the theory of traditional Chinese medicine, “Lian Zi Xin” has been applied in cardiovascular diseases such as hypertension, arrhythmia, and hyperlipidemia (Qian, 2002). Isoliensinine, a prominent alkaloid derived from “Lian Zi Xin,” also possesses cardiovascular protective activities, such as exerting anti-arrhythmia and antihypertension effects and ameliorating ventricular hypertrophy (Table 2).

**TABLE 2:** Cardiovascular protective activity of isoliensinine.

Effect	Model	Dose	Mechanism	Result	References
Reverse cerebral ischemic damage	Rats	5–20 mg/kg	MDA↓, ROS↓, SOD↑, ATPase↑	Oxidative stress↓, [Ca^2+^] ↓	Xu et al. (2005)
Hypotensive	Rats	2.5–10 mg/kg	α _1_↓, VGCCs↓	The aortic ring contraction response↓	Zhang et al. (2000)
	CASMs	0.01–3 μM	PDGF-β↓, bFGF↓, c-fos↓, c-myc↓, hsp70↓	Proliferation of CASMs↓	Xiao et al. (2005), Xiao et al. (2006)
	Rats VSMCs	50–200 μg/ml	Collagen I↓, α-SMA↑, *p*-MYPT1↓, ROCK/RhoA↓	Ameliorating aortic remodeling; vasodilatation	Li et al. (2019)
Improve left ventricular hypertrophy	Rats	20 mg/kg	α_1_ receptor↓, Ca^2+^ channel↓, SERCA↑	Left atrial contractility↓	Feng et al. (2002); Lu et al. (2008)
				Right ventricular self-pacing frequency↓	
				Blood pressure↓	
Anti-arrhythmia	Rabbit, rats, Guinea pig	1.25–2.5 mg/kg	Ca^2+^ channel↓, Na^+^ channel↓	APA↓, Vmax↓, APD↓, EADs↓, DADs↓, I _NaL_↓, I _CaL_↓	Zhang et al. (2000); Liu et al. (2020)
Protecting vascular endothelial cells	ECV304	0.1 μM	NOS↑, NO↑	Cell survival↑	Zang et al. (2015)

↓, downregulation, inactivation, inhibition; ↑, upregulation, activation.

#### Antihypertension and Amelioration of Left Ventricular Hypertrophy

Antihypertensive drugs are divided into the following types according to their hypotensive mechanisms: Ca^2+^ channel blockers, ß-receptor blockers, angiotensin-converting enzyme inhibitors (ACEIs), angiotensin receptor blockers (ARBs), and diuretics (Laurent, 2017). Isoliensinine had a transient, dose-dependent antihypertensive effect on anesthetized rats. It inhibited the aortic ring contraction response induced by methoxamine and high K^+^, which indicated that isoliensinine blocked the α_1_ receptor, thereby inhibiting the release of internal calcium and the influx of external calcium caused by the α_1_ receptor. It also blocked voltage-dependent calcium channel activity (Zhang et al., 2000a). Angiotensin II (Ang II) is the major effective peptide of the renin–angiotensin system, which plays an important role in several cardiovascular diseases associated with vascular smooth muscle cell (VSMC) overgrowth and inflammation, including hypertension, atherosclerosis, and myocardial infarction. Phenylephrine, a powerful vasoconstrictor, causes high blood pressure. As Xiao et al. reported, 0.01–3 μmol/l isoliensinine markedly blocked the proliferation of porcine coronary arterial smooth muscle cells induced by angiotensin II and phenylephrine (Xiao et al., 2005a; Xiao et al., 2006). Mechanistically, isoliensinine significantly inhibited the overexpression of basic fibroblast growth factor (bFGF) and platelet-derived growth factor (PDGF-β) and decreased the overexpression of the proto-oncogenes c-fos, c-myc, and hsp70 evoked by angiotensin II and phenylephrine. Interestingly, the inhibitory effect of isoliensinine on PDGF-β was even stronger than that of irbesartan. In an 8-week-old healthy male or female C57BL/6 J mouse model, isoliensinine was demonstrated to be a potential relaxant for abnormal isolated smooth muscle contractions induced by KCl with IC_50_ values of 3.504 μM (Yang et al., 2018). Recently, alkaloids from *Nelumbinis plumula* (AFNP), including isoliensinine, liensinine, and neferine, displayed excellent antihypertension and aortic protection effects *via* nonendothelial-dependent inhibition of VSMC cytoskeleton remodeling and regulation of the RhoA/ROCK pathway (Li et al., 2019). On the one hand, AFNP exhibited a strong vasodilatation effect on Ang II–induced rat aortic tissue constriction by inhibiting the proliferation of VSMCs. On the other hand, it also normalized the effects of hypertension in male SHRs by reversing RhoA transposition, ROCK activation, MYPT1 phosphorylation, and Ang II–induced collagen upregulation and increasing α-SMA expression.

One of the common causes of left ventricular hypertrophy is chronic stress overload of the heart, which can subsequently accelerate the progression of arrhythmia, heart failure, and other fatal cardiovascular diseases (Yildiz et al., 2020). Compared with normal saline, isoliensinine (10–20 mg/kg) dose dependently inhibited left atrial contractility and right atrial self-beating frequency and shifted the Ca^2+^ dose-effect curve to the right in a rat model of left ventricular hypertrophy. Additionally, isoliensinine reversed the effect of phenylephrine on the blood pressure of rats with spinal cord damage. It significantly weakened the changes in vascular pressure caused by partial aortic stenosis and reduced the weight of the heart. All of these changes suggest that isoliensinine noncompetitively inhibits calcium influx, blocking α_1_ receptors and reducing left ventricular hypertrophy (Feng et al., 2002). Another study also confirmed that isoliensinine improved the activity of the myocardial calcineurin calcium pump, alleviated intracellular calcium overload, and reduced blood pressure, which subsequently reversed left ventricular hypertrophy (Lu et al., 2008).

#### Anti-Arrhythmia

An arrhythmia model was constructed through the induction of drugs (ouabain, aconitine, calcium chloride, and epinephrine) and coronary artery ligation. Zhang et al. proved that isoliensinine reduced arrhythmias and that its effect was stronger than that of quinidine. The study showed that isoliensinine reduced APA and Vmax and shortened APD, suggesting that its mechanism was related to blocking Ca^2+^ and Na^+^ currents (Zhang et al., 2000b). Isoliensinine also had an inhibitory effect on stretch arrhythmias, and this effect was also achieved by blocking the ion channels activated by stretch (Chen et al., 2006). Moreover, Liu et al. (Liu et al., 2020) showed that isoliensinine eliminated afterdepolarizations by inhibiting late sodium current (*I*_NaL_) and L-type calcium current (*I*_CaL_). The late sodium current is caused by the slow inactivation of some sodium channels after a large number of sodium channels are rapidly inactivated and is considered a target of new antiarrhythmic drugs (Antzelevitch et al., 2014). The L-type calcium current and late sodium current participate in the formation of the AP plateau. Isoliensinine effectively eliminated early afterdepolarizations and delayed afterdepolarizations induced by ATX-II by inhibiting *I*_NaL_ and *I*_CaL_ rather than the inward rectifier potassium current (*I*_K1_) and delayed rectifier potassium current in isolated rabbit left ventricular myocytes, which indicates that isoliensinine has potent antiarrhythmic activity.

#### Reversal of Cerebral Ischemic Damage

In an ischemia/reperfusion injury model induced by bilateral common carotid artery occlusion and hypotension, isoliensinine (5–20 mg/kg) increased SOD and ATPase activity and decreased the MDA content and LDH activity in brain tissue and brain cell mitochondria (Xu et al., 2005). A later study confirmed that intervention with 3 mg/kg liensinine in a rat focal ischemia model also downregulated the expression of the c-Fos protein and exerted a protective effect against cerebral ischemia (Yu et al., 2007).

#### Other Effects

In addition to the pharmacological effects compiled above, isoliensinine also possesses antiplatelet aggregation and anticoagulation effects. Isoliensinine inhibited the aggregation rate of platelets in rabbits induced by adenosine diphosphate *in vivo* and *in vitro* and increased the values of PT, APTT, and TT in rats (Chen and Luo, 2011). It also had a protective effect against H_2_O_2_-induced oxidative damage in ECV304 human umbilical vein endothelial cells (Zang et al., 2015). Isoliensinine had no effect on normal ECV304 cells, but it alleviated the H_2_O_2_-induced inhibition of the activity of ECV304 human umbilical vein endothelial cells and reversed the abnormal morphological changes. Mechanistically, isoliensinine increases the generation of nitric oxide synthase (NOS) to increase the release of nitric oxide (NO) from vascular endothelial cells, thus protecting the endothelium.

### Antioxidant Activity

Maintaining redox system homeostasis is vital to maintain the health of the body. Therefore, if the redox system is out of balance, it will lead to a variety of injuries and damage to the body (Shadel and Horvath, 2015). For example, the excessive generation of ROS induces oxidative stress and damages various necessary molecules in cells, such as DNA, proteins, and lipids, thereby inducing rapid cell senescence and even death (Apel and Hirt, 2004; Gorrini et al., 2013). In other words, antioxidation plays a key role in resisting the aging process (Liochev, 2013). Shen et al. (Shen et al., 2017) summarized a series of ingredients from herbs and nutraceuticals for antiaging that are used in traditional Chinese medicine, including isoliensinine. Compared with the model group of D-galactose–induced aging mice, the isoliensinine group exhibited significantly reduced MDA contents in serum and liver tissues and increased activities of SOD and GSH-Px (Liu et al., 2011). Other studies have also obtained similar results. Xie et al. (Xie et al., 2013) confirmed that *Nelumbinis Plumula* total alkaloid (NPA) had a protective effect against the oxidative stress damage of HepG2 cells induced by tert-butyl hydroperoxide. The EC_50_ value of the total alkaloids to clear DPPH was 82.56 μg/ml. NPA reduced the tert-butyl hydroperoxide–induced upregulation of MDA, ROS, and LDH and upregulated GSH-Px, which was inhibited by tert-butyl hydroperoxide. The researchers then identified the main components of total alkaloids derived from the heart of *Nelumbinis*, which primarily included liensinine 2.57%, isoliensinine 4.38%, and neferine 9.25%. Moreover, isoliensinine also exerted protective effects against cerebral ischemic damage and kidney damage caused by high glucose by inhibiting oxidative stress. The effect of isoliensinine on ischemic cerebrovascular injury can be found in *anti-hypertension and amelioration of left ventricular hypertrophy*. Isoliensinine had a protective effect in the kidney predominantly by weakening the activity of NADPH oxidase (Deng, 2015). Diabetic rats induced by streptozotocin for 72 h were treated with isoliensinine (20–200 mg/kg), and the kidney tissues of the sacrificed rats were used for pathological sections and immunoblotting after 8 weeks. In diabetic rats, compared with the control group, the isoliensinine-treated group exhibited reduced kidney tissue changes in a dose-dependent manner, including weakened NADPH oxidase activity and decreased expression of the NADPH oxidase subunits p22phox and p67phox, as indicated by Western blotting. Primary glomerular cells were isolated and cultured for 20 days. They were treated with isoliensinine (5–40 μM) for 1 and 12 h after stimulation with high glucose. The results obtained with immunofluorescence probes showed that the NADPH oxidase activity was weakened after treatment with isoliensinine for 1 h.

### Anti-HIV Activity

Human immunodeficiency virus (HIV) is very harmful to humans. Once an individual is infected with HIV, it will continue to attack the body’s immune system. After a prolonged asymptomatic period of years to decades, it will lead to lethal AIDS. Various complications characterize AIDS, including wasting, neurological impairment, opportunistic infections, and malignancies (Richman, 2001). Studies have found that alkaloids isolated from *Nelumbo nucifera* possess efficient anti-HIV activity. Kashiwad et al. (Kashiwada et al., 2005) found that (+)-1(*R*)-coclaurine and (−)-1(*S*)-norcoclaurine extracted from lotus leaves have potent anti-HIV activities with EC_50_ values of 0.8 μg/ml and <0.8 μg/ml, respectively, and TI values (the ratio of IC_50_ to EC_50_) of >125 and >25, respectively. Structure–activity analysis led them to speculate that isoliensinine has a similar activity. Then, they proved the anti-HIV activity of isoliensinine with EC_50_ <0.8, IC_50_ <5.17, and TI>0.6 by building an H9 cell model infected with HIV-1 and measuring p24 antigen levels. Later, Zhou et al. (Zhou et al., 2007) also determined that alkaloids with bisbenzylisoquinoline structures, such as isoliensinine and its analogs, have strong anti-HIV activities. In summary, isoliensinine and its analogs can serve as lead compounds for the further development of anti-AIDS agents.

### Anti–type 2 Diabetes Activity

The primary cause of T2DM is obesity-driven insulin resistance in the liver, white adipose tissue, and skeletal muscle, combined with secretion of insulin by pancreatic ß cells that is insufficient to overcome this resistance (Chatterjee et al., 2017). Yang et al. (Yang et al., 2017) confirmed that isoliensinine effectively reduced the positive symptoms of T2DM with hyperlipidemia by using L6 rat skeletal muscle cells and the KK-Ay rat model with metabolic disorders. They then investigated whether isoliensinine elevated the expression of GLUT4, promoted the translocation of GLUT4 to the plasma membrane, and evoked the uptake of blood sugar. However, the stimulation of GLUT4 induced by isoliensinine was completely inhibited by compound C, an inhibitor of AMPK. Isoliensinine treatment also significantly reduced the body weight and blood glucose level and improved the OGTT compared with vehicle treatment. In addition, isoliensinine decreased the overexpression of the lipogenesis proteins PPARY, SREBP-1C, and P-ACC/ACC in KK-Ay rats. The immunohistochemical results of liver and white fat also showed the hypoglycemic and blood lipid-lowering effects of isoliensinine.

### Central Neuroprotection

Inhibition of cholinesterases (ChEs) to increase the amount of acetylcholine (ACh) has been acknowledged as the most effective treatment against Alzheimer’s disease (AD) since cholinergic projection is considered to be a major mechanism of AD pathogenesis (Isacson et al., 2002; Barnett, 2019). Acetylcholinesterase (AChE) and butyrylcholinesterase (BChE), which coexist in the central nervous system (CNS), are capable of efficiently hydrolyzing ACh at different rates, which indicates that BChE is also a therapeutic target (Darvesh, 2016; Sharma et al., 2017). As Lin et al. (Lin et al., 2013) reported isoliensinine is a potential drug candidate for the treatment of AD. They first determined the biological activity of alkaloids derived from lotus seeds possessing a strong inhibitory effect on BChE by building an on-line biochemical detection method. In particular, the tacrine-equivalent doses of the three alkaloids liensinine, isoliensinine, and neferine were 58.25 ± 2.16 μmol/g, 16.81 ± 0.77 μmol/g, and 46.81 ± 2.09 μmol/g, respectively, which indicated that isoliensinine had the strongest inhibitory effect on BChE among the three alkaloids. Isoliensinine also exhibits potent anti-amnestic activity (Zhang, 2010). The enzyme activity test proved that isoliensinine possessed an inhibitory effect on acetylcholinesterase and butyrylcholinesterase in rat brain tissue and plasma with IC_50_ values of 6.82 ± 0.25 μM and 15.51 ± 2.20 μM, respectively. In the Morris water maze task, the latency to the first target-site crossover in the model and isoliensinine (1, 10, 30 mg/kg) groups were 20.59 ± 1.40 s, 16.54 ± 2.72 s, 16.43 ± 2.06 s, and 14.45 ± 2.47 s, respectively. In the Y maze task, the alternation rates of the model and isoliensinine (30 mg/kg) groups were 34.67 ± 1.96% and 44.74 ± 2.23%, respectively. Behavioral experiments also confirmed that isoliensinine had a stronger therapeutic effect on learning and memory disorders in mice. PC12 cells were treated with Aβ_25–35_ or isoliensinine (0.1–10 μM) alone or in combination, and it was found that isoliensinine treatment alone had no impact on PC12 cells, while Aβ_25–35_ alone decreased PC12 cell numbers and changed PC12 cell morphology, leading to irregular spindle shape, pyknosis, and gap changes. After cotreatment, isoliensinine restored the survival rate of PC12 cells downregulated by Aβ_25–35_ and suppressed the morphological changes induced by Aβ_25–35_. Mechanistically, isoliensinine downregulated Ca^2+^ concentration and the production of ROS induced by Aβ_25–35_. Isoliensinine decreased the hyperphosphorylation of Tau caused by Aβ_25–35_, thereby reducing the expression of intracellular CaM. APP/PS1 double transgenic AD mice were treated with total alkaloids from the seed embryo of *Nelumbo nucifera* (TASENN) for 28 days and then sacrificed. Brain tissues were taken for pathological and Western blotting experiments. Observation of mouse brain tissue with HE staining showed that the nuclei of pyramidal cells of APP/PS1 transgenic AD mice were deeply stained and condensed, and the cells were triangular or irregular in shape. The hippocampal pyramidal cells of the drug-administered group were regular in shape and densely arranged, and the nuclei were large and round and showed uniform light staining. TUNEL staining showed that the number of apoptotic neuronal cells in the mouse brain decreased after TASENN administration, indicating that the drug inhibited mouse neuronal cell apoptosis. The Western blot results proved that TASENN significantly inhibited the abnormal phosphorylation of Tau protein in the hippocampus and cortex of APP/PS1 transgenic AD mice and reduced the expression of CaM (Gou, 2019).

Recently, the number of people with depression has increased with faster pace of society and increase in life pressure (Malhi and Mann, 2018). In 2015, Japanese scholars Sugimoto et al. (Sugimoto et al., 2015) demonstrated that alkaloids isolated from “Lian Zi Xin” elicited potential antidepressant effects. They found that isoliensinine at 25 mg/kg and 50 mg/kg apparently reduced the immobility time of mice in the forced swimming test. Interestingly, the 5-HT_1A_ receptor antagonist WAY 106335 reversed the effects of isoliensinine.

### Protective Effect on Respiratory System

Paraquat (PQ) is an effective and widely used herbicide. However, it leads to fatal damage by accumulating in the lungs after excessive intake (Dinis-Oliveira et al., 2008). Isoliensinine has been reported to reduce PQ-induced acute lung injury. Mechanistically, isoliensinine augmented SOD activity and downregulated MDA content and ALP activity induced by paraquat. In addition, it also reduced the overexpression of TGF-β1 and MMP2 caused by PQ (Tang et al., 2007).

Bleomycin is commonly used in the clinic to treat various squamous-cell carcinomas, including cervical cancer, esophageal cancer, external genital cancer, and head–neck cancer. However, it often leads to a large number of adverse reactions, such as severe pulmonary fibrosis and skin diseases (Dirix et al., 1994; Brandt and Gerriets, 2020). Xiao et al. (Xiao et al., 2005b) found that isoliensinine significantly alleviated bleomycin-induced pulmonary fibrosis in Kunming mice. The study demonstrated that isoliensinine dramatically suppressed the excessive increase in MDA and hydroxyproline induced by bleomycin in lung tissue and serum in a concentration-dependent manner compared with normal saline and increased the activity of SOD, which was decreased by bleomycin. Moreover, isoliensinine inhibited the overexpression of TGF-β1 and TNF-α induced by bleomycin. In a high K^+^–induced isolated mouse airway smooth muscle (ASM) model, isoliensinine inhibited L-shaped voltage-dependent Ca^2+^ channel (LVDCC) currents, terminated Ca^2+^ influx, and reduced [Ca^2+^]_i_, eventually resulting in the relaxation of the ASM, which indicates that isoliensinine is a potential bronchodilator (Liu et al., 2019).

## Conclusions and Prospects

As described above, isoliensinine has displayed curative effects in numerous diseases *in vitro* and *in vivo*. First, isoliensinine exhibits potent effects on various tumor cells. As a small molecule inducer, isoliensinine induces the autophagic death of HeLa cervical cancer cells and apoptosis-resistant MEFs by activating the AMPK–TSC2–mTOR pathway. For triple-negative breast cancer, isoliensinine possesses the strongest inhibitory activity on MDA-MB-321 cells among isoliensinine, liensinine, and neferine, and it selectively inhibits growth and induces apoptosis by increasing ROS generation and activating P38MAPK/JNK. Shu et al. confirmed that isoliensinine had a strong toxic effect on hepatocellular carcinoma both *in vivo* and *in vitro*. However, isoliensinine did not show obvious toxic effects in the liver, spleen, or lung, as indicated by organic indexes, compared with saline in tumor-bearing Kunming mice. In addition, isoliensinine promoted the uptake of cisplatin and sensitized HCT-15 colorectal cancer cells to the inhibitory effect of cisplatin. In addition to its toxic effect on tumor cells, isoliensinine has also been demonstrated to exert protective effects on cells in other important organs of the body. Isoliensinine has shown excellent cardiovascular protection abilities, including anti-hypertension and anti-arrhythmia effects, and has been shown to inhibit coronary artery remodeling. Additionally, it exhibits beneficial effects in the nervous system, including ameliorating Alzheimer’s disease by inhibiting BChE activity and alleviating depression by acting on 5-HT_1A_ receptors. Moreover, isoliensinine also efficiently resists oxidative damage by reducing the levels of MDA and ROS as well as increasing SOD activity and exhibits a series of beneficial effects, such as antiaging effects. Isoliensinine was shown to reverse the adverse effects of the anticancer drug bleomycin in pulmonary fibrosis and to elevate the ability of the immune system to respond to HIV infection. The multi-pharmacological activity of isoliensinine confirmed by existing studies indicates that it is indeed a great drug candidate for the development of new drugs for various diseases.

Isoliensinine is primarily derived from “Lian Zi Xin,” the green embryo of the lotus seed, which is often used as an ingredient in everyday foods, such as tea and soup, in Asia. Thus, we can conclude that isoliensinine is a safe candidate for screening “drug-like” compounds. It is no longer difficult to obtain high-purity isoliensinine after scholars have worked hard to improve the separation and purification methods in the past 2 decades. Although isoliensinine can also be synthesized chemically, the specific biosynthetic pathways are not well understood. The *in vivo* metabolic process of isoliensinine has also been clarified in mice and beagle dogs. Isoliensinine is mainly metabolized by the liver, and three metabolites are generated *via N*-demethylation and *O*-demethylation, including 2-*N*-desmethyl-isoliensinine, 2′-*N*-desmethylisoliensinine, and 2′-*N*-6-*O*-didesmethylisoliensinine. Nevertheless, it has been proven that isoliensinine does not interact with the liver microsomal enzyme CYP3A4, but the specific CYP450 enzymes that interact with isoliensinine are not clear.

Indeed, isoliensinine displays a great deal of advantageous therapeutic effects. However, apart from seven reports on the antitumor activity of isoliensinine, there are few reports of other pharmacological activities of isoliensinine, and the related mechanisms are not specific enough. More detailed experiments are needed. Notably, the activity of isoliensinine is at the micromolar level in available reports. Moreover, isoliensinine has poor solubility in the aqueous medium, which will seriously affect its absorption rate in the body and thus affect its clinical efficacy. Accordingly, medicinal chemists must modify its structure according to the structure-activity relationship to ameliorate the lipo–hydro partition coefficient of isoliensinine. In addition, its solubility can also be increased by changing the crystal form and preparing liposomes, nanoparticles, microspheres, microemulsions, and water-soluble precursors. Most importantly, experimental studies on isoliensinine have mainly focused on cells, so more animal experiments and clinical experiments are needed to further investigate the medicinal value of isoliensinine.

In summary, the available evidence verifies that isoliensinine is a natural compound that possesses a number of beneficial effects against many diseases, and it is safer than many chemically synthesized substances since it originates from an edible plant. In future research, high-throughput sequencing and computer molecular target predictions can be used to identify more active targets of isoliensinine that have not yet been reported. In summary, isoliensinine can be used as a lead compound to obtain more compounds with greater curative effects, lower required doses, less toxicity, fewer side effects, and more suitable lipo–hydro partition coefficients *via* chemical structure modification and other optional reasonable decoration approaches.

## GLOSSARY

α-SMA: Alpha smooth muscle actin
ACC: acetyl CoA carboxylase
AIDS: acquired immunodeficiency syndrome
AKT: protein kinase B
AMPK: 5-AMP activated protein kinase
AP: action potential
APTT: activated partial thromboplastin time
BCRP: breast cancer resistance protein
bFGF: basic fibroblast growth factor
CASMs: coronary arterial smooth muscle cells
DPPH: 1,1-diphenyl-2-picrylhydrazyl
EC50: concentration for 50% of maximal effect
GLUT4: glucose transporter 4
GSH-Px: glutathione peroxidase
HK2: hexokinase 2
IC_50_: half maximal inhibitory concentration
I2PP2A: inhibitor-2 of protein phosphatase-2A
LDH: lactic dehydrogenase
MAPK: mitogen-activated protein kinase
MDA: malondialdehyde
MEFs: mouse embryonic fibroblasts
MMP: mitochondrial membrane potential
MRP2: multidrug-resistant associated protein 2
mTOR: mammalian target of rapamycin
MYPT1: myosin phosphatase (phospho T853)
OGTT: oral glucose tolerance
PI3K: phosphatidylinositol 3-kinase
PDGF-β: platelet-derived growth factorβ
PKM2: pyruvate kinase M2
PP2A: protein phosphatase 2A
PPARY: adipogenic transcription factor
PT: prothrombin time
RhoA: small guanosine triphosphate binding protein Ras homolog gene family member A
ROCK: Rho-associated kinase;
SOD: superoxide dismutase
SREBP-1C: rat sterol regulatory element-binding protein 1C
ROS: reactive oxygen species
T2DM: type 2 diabetes
TGF-β1: transforming growth factor-β1
TI: the ratio of IC50 to EC50
TNF-α: tumor necrosis factor-α
TNBSs: triple-negative breast cancer cell
TSC2: tuberous sclerosis 2
TT: thrombin time
TUNEL: TdT-mediated dUTP nick-end labeling
Vd: apparent volume of distribution
VGCCs: voltage-gated Ca2+ channels
VSMCs: vascular smooth muscle cells

## References

[B1] Abu Samaan T. M. Samec M. Liskova A. Kubatka P. Büsselberg D. (2019). Paclitaxel's Mechanistic and Clinical Effects on Breast Cancer. Biomolecules 9 (12), 789. 10.3390/biom9120789 PMC6995578 31783552

[B2] Antzelevitch C. Nesterenko V. Shryock J. C. Rajamani S. Song Y. Belardinelli L. (2014). The Role of Late I Na in Development of Cardiac Arrhythmias. Handb Exp. Pharmacol. 221, 137–168. 10.1007/978-3-642-41588-3_7 24737235 PMC4076160

[B3] Apel K. Hirt H. (2004). Reactive Oxygen Species: Metabolism, Oxidative Stress, and Signal Transduction. Annu. Rev. Plant Biol. 55, 373–399. 10.1146/annurev.arplant.55.031903.141701 15377225

[B4] Ashley E. A. Pyae Phyo A. Woodrow C. J. (2018). Malaria. Lancet 391 (10130), 1608–1621. 10.1016/s0140-6736(18)30324-6 29631781

[B5] Barnett R. (2019). Alzheimer's Disease. Lancet 393 (10181), 1589. 10.1016/s0140-6736(19)30851-7 31007191

[B6] Brandt J. P. Gerriets V. (2020). Bleomycin. StatPearls. Treasure Island (FL): StatPearls Publishing LLC.

[B7] Chao T. Y. Chou Y. L. Young P. T. Chou T. Q. (1962). Studies on the Alkaloids of Embryo Loti, *Nelumbo nucifera* Gaertn. I. Isolation and Characterisation of Liensinine. Sci. Sin 11, 215–219. PMID: 13878148. 13878148

[B8] Chatterjee S. Khunti K. Davies M. J. (2017). Type 2 Diabetes. Lancet 389 (10085), 2239–2251. 10.1016/s0140-6736(17)30058-2 28190580

[B9] Chen G. Zhu M. Guo M. (2019). Research Advances in Traditional and Modern Use of *Nelumbo nucifera*: Phytochemicals, Health Promoting Activities and beyond. Crit. Rev. Food Sci. Nutr. 59 (Suppl. 1), S189–S209. 10.1080/10408398.2018.1553846 30633540

[B10] Chen J. P. Li X. Y. (2011). Pharmacokinetics Study of Isoliensine in Rats. Chin. Pharm. 22 (23), 2130–2132. CNKI:SUN:ZGYA.0.2011-23-012

[B11] Chen L. J. Luo D. S. (2011). Isoliensinine's Antiplatelet Aggregation and Anticoagulant Effects. Chin. Hosp. Pharm. 31 (15), 1257–1259. CNKI:SUN:ZGYZ.0.2011-15-010

[B12] Chen S. M. Jia L. Fei Y. J. Ma R. Yang L. Wang J. L. (2006). Effect of Isoliensinine on Stretch-Induced Arrhythmia. Chin. Hosp. Pharm. 26 (06), 658–661.

[B13] Chen Y. Fan G. Wu H. Wu Y. Mitchell A. (2007). Separation, Identification and Rapid Determination of Liensine, Isoliensinine and Neferine from Embryo of the Seed of *Nelumbo nucifera* Gaertn. by Liquid Chromatography Coupled to Diode Array Detector and Tandem Mass Spectrometry. J. Pharm. Biomed. Anal. 43 (1), 99–104. 10.1016/j.jpba.2006.06.016 16846715

[B14] Commission N. P. (2015). The Pharmacopoeia of the People’s Republic of China. Chin Pharmacop Commit 1: 273–274. 10.1002/minf.201700048

[B15] Daina A. Michielin O. Zoete V. (2017). SwissADME: a Free Web Tool to Evaluate Pharmacokinetics, Drug-Likeness and Medicinal Chemistry Friendliness of Small Molecules. Sci. Rep. 7, 42717. 10.1038/srep42717 28256516 PMC5335600

[B16] Darvesh S. (2016). Butyrylcholinesterase as a Diagnostic and Therapeutic Target for Alzheimer's Disease. Car 13 (10), 1173–1177. 10.2174/1567205013666160404120542 27040140

[B17] Deng B. (2015). Study on the Protective Effect and Mechanism of Lotus Seed Heart Extract on the Kidney of Diabetic Rats Master's Degree. Chengdu, Sichuan, China: Chengdu Medical College.

[B18] Diamond A. Desgagné-Penix I. (2016). Metabolic Engineering for the Production of Plant Isoquinoline Alkaloids. Plant Biotechnol. J. 14 (6), 1319–1328. 10.1111/pbi.12494 26503307 PMC11389028

[B19] Dinis-Oliveira R. J. Duarte J. A. Sánchez-Navarro A. Remião F. Bastos M. L. Carvalho F. (2008). Paraquat Poisonings: Mechanisms of Lung Toxicity, Clinical Features, and Treatment. Crit. Rev. Toxicol. 38 (1), 13–71. 10.1080/10408440701669959 18161502

[B91] Dirix L. Y. Schrijvers D Druwé P Van den Brande J Verhoeven D Van Oosterom A. T. (1994). Pulmonary toxicity and bleomycin. Lancet 344 (8914), 56. 10.1016/s0140-6736(94)91076-6 7516990

[B20] Do T. C. M. V. Nguyen T. D. Tran H. Stuppner H. Ganzera M. (2013). Analysis of Alkaloids in Lotus (*Nelumbo nucifera* Gaertn.) Leaves by Non-aqueous Capillary Electrophoresis Using Ultraviolet and Mass Spectrometric Detection. J. Chromatogr. A 1302, 174–180. 10.1016/j.chroma.2013.06.002 23838305

[B21] Duanmu Q. Li A. Sun A. Liu R. Li X. (2010). Semi-preparative High-Speed Counter-current Chromatography Separation of Alkaloids from Embryo of the Seed of *Nelumbo nucifera* Gaertn by pH-Gradient Elution. J. Sep. Sci. 33 (12), 1746–1751. 10.1002/jssc.200900872 20437410

[B22] Facchini P. J. De Luca V. (2008). Opium Poppy and Madagascar Periwinkle: Model Non-model Systems to Investigate Alkaloid Biosynthesis in Plants. Plant J. 54 (4), 763–784. 10.1111/j.1365-313X.2008.03438.x 18476877

[B23] Fang Y. Li Q. Shao Q. Wang B. Wei Y. (2017). A General Ionic Liquid pH-Zone-Refining Countercurrent Chromatography Method for Separation of Alkaloids from *Nelumbo nucifera* Gaertn. J. Chromatogr. A 1507, 63–71. 10.1016/j.chroma.2017.05.048 28571916

[B24] Feng X. L. Yu X. Xiao J. H. Wang J. L. (2002). Effect of Isoliensinine on Cardiovascular Function and Experimental Left Ventricular Hypertrophy. Huazhong Univ. Sci. Tech. [ Health Sci. ] 31 (06), 608–611. CNKI:SUN:TJYX.0.2002-06-002

[B25] Forner A. Reig M. Bruix J. (2018). Hepatocellular Carcinoma. Lancet 391 (10127), 1301–1314. 10.1016/s0140-6736(18)30010-2 29307467

[B26] Glenn W. S. Runguphan W. O’Connor S. E. (2013). Recent Progress in the Metabolic Engineering of Alkaloids in Plant Systems. Curr. Opin. Biotechnol. 24 (2), 354–365. 10.1016/j.copbio.2012.08.003 22954587 PMC3552043

[B27] Gorrini C. Harris I. S. Mak T. W. (2013). Modulation of Oxidative Stress as an Anticancer Strategy. Nat. Rev. Drug Discov. 12 (12), 931–947. 10.1038/nrd4002 24287781

[B28] Gou J. M. (2019). Protective Effects of Alkaloids of Plumula Nelumbinis on Aβ-Induced PC12 Cells Injury and Their Anti-Alzheimer's Disease Effect Master's Degree. Liaoning, China: Shenyang Liaoning University.

[B29] Hu G. Xu R.-A. Dong Y.-Y. Wang Y.-Y. Yao W.-W. Chen Z.-C. (2015). Simultaneous Determination of Liensinine, Isoliensinine and Neferine in Rat Plasma by UPLC-MS/MS and Application of the Technique to Pharmacokinetic Studies. J. Ethnopharmacol. 163, 94–98. 10.1016/j.jep.2015.01.020 25636663

[B30] Huang Y. Bai Y. Zhao L. Hu T. Hu B. Wang J. (2007). Pharmacokinetics and Metabolism of Neferine in Rats after a Single Oral Administration. Biopharm. Drug Dispos. 28 (7), 361–372. 10.1002/bdd.556 17654697

[B31] Huang Y. Zhao L. Bai Y. Liu P. Wang J. Xiang J. (2011). Simultaneous Determination of Liensinine, Isoliensinine and Neferine from Seed Embryo of *Nelumbo nucifera* Gaertn. In Rat Plasma by a Rapid HPLC Method and its Application to a Pharmacokinetic Study. Arzneimittelforschung 61 (6), 347–352. 10.1055/s-0031-1296209 21827045

[B32] Isacson O. Seo H. Lin L. Albeck D. Granholm A. C. (2002). Alzheimer's Disease and Down's Syndrome: Roles of APP, Trophic Factors and ACh. Trends Neurosci. 25 (2), 79–84. 10.1016/s0166-2236(02)02037-4 11814559

[B33] Jaiswal Y. Liang Z. Zhao Z. (2016). Botanical Drugs in Ayurveda and Traditional Chinese Medicine. J. Ethnopharmacol. 194, 245–259. 10.1016/j.jep.2016.06.052 27394388

[B34] Jin Y. Khadka D. B. Cho W.-J. (2016). Pharmacological Effects of Berberine and its Derivatives: a Patent Update. Expert Opin. Ther. Patents 26 (2), 229–243. 10.1517/13543776.2016.1118060 26610159

[B35] Kametani T. Takano S. Iida H. Shinbo M. (1969). A Total Synthesis of Isoliensinine. J. Chem. Soc. C 1 2, 298–300. 10.1039/j39690000298 5814734

[B36] Kashiwada Y. Aoshima A. Ikeshiro Y. Chen Y.-P. Furukawa H. Itoigawa M. (2005). Anti-HIV Benzylisoquinoline Alkaloids and Flavonoids from the Leaves of *Nelumbo nucifera*, and Structure-Activity Correlations with Related Alkaloids. Bioorg. Med. Chem. 13 (2), 443–448. 10.1016/j.bmc.2004.10.020 15598565

[B37] Katz L. Baltz R. H. (2016). Natural Product Discovery: Past, Present, and Future. J. Ind. Microbiol. Biotechnol. 43 (2-3), 155–176. 10.1007/s10295-015-1723-5 26739136

[B38] Laurent S. (2017). Antihypertensive Drugs. Pharmacol. Res. 124, 116–125. 10.1016/j.phrs.2017.07.026 28780421

[B39] Law B. Y. K. Chan W. K. Xu S. W. Wang J. R. Bai L. P. Liu L. (2014). Natural Small-Molecule Enhancers of Autophagy Induce Autophagic Cell Death in Apoptosis-Defective Cells. Sci. Rep. 4, 5510. 10.1038/srep05510 24981420 PMC4076737

[B40] Li W. Qiu Y. Hao J. Zhao C. Deng X. Shu G. (2018). Dauricine Upregulates the Chemosensitivity of Hepatocellular Carcinoma Cells: Role of Repressing Glycolysis *via* miR-199a:HK2/PKM2 Modulation. Food Chem. Toxicol. 121, 156–165. 10.1016/j.fct.2018.08.030 30171973

[B41] Li Q. Wo D. Huang Y. Yu N. Zeng J. Chen H. (2019). Alkaloids from Nelumbinis Plumula (AFNP) Ameliorate Aortic Remodeling *via* RhoA/ROCK Pathway. Biomed. Pharmacother. 112, 108651. 10.1016/j.biopha.2019.108651 30784931

[B42] Lin Z. Wang H. Fu Q. An H. Liang Y. Zhang B. (2013). Simultaneous Separation, Identification and Activity Evaluation of Three Butyrylcholinesterase Inhibitors from Plumula Nelumbinis Using On-Line HPLC-UV Coupled with ESI-IT-TOF-MS and BChE Biochemical Detection. Talanta 110, 180–189. 10.1016/j.talanta.2013.02.033 23618192

[B43] Lin Z. Zhang C. Cao D. Damaris R. N. Yang P. (2019). The Latest Studies on Lotus (*Nelumbo nucifera*)-an Emerging Horticultural Model Plant. Int. J. Mol. Sci. 20 (15), 3680. 10.3390/ijms20153680 PMC6696627 31357582

[B92] Liochev S. I. (2013). Reactive oxygen species and the free radical theory of aging. Free Radic Biol Med 60, 1–4. 10.1016/j.freeradbiomed.2013.02.011 23434764

[B44] Liu S. L. Hao Y. R. Qin J. Yu L. (2011). Study on Antioxidation of Isoliensinine in D-Galactose-Induced Agedmice. China Med. Herald 8 (20), 5–6+9. 10.3969/j.issn.1673-7210.2011.20.002

[B45] Liu B. B. Chen W. W. Zhang W. J. Liu Q. H. (2019). Relaxation Effect of Isoliensinine on Isolated Mouse Airway Smooth Muscle. Chin. J. Pathophysiol. 35 (05), 920–925. CNKI:SUN:ZBLS.0.2019-05-023

[B46] Liu Z. Hu L. Zhang Z. Song L. Zhang P. Cao Z. (2020). Isoliensinine Eliminates Afterdepolarizations through Inhibiting Late Sodium Current and L-type Calcium Current. Cardiovasc. Toxicol. 21, 67–68. 10.1007/s12012-020-09597-z 32770463

[B47] Lu S. Zhang Z. B. Gong S. Y. Su W. Zhu H. J. (2008). Possible Mechanism of Isoliensinine Preventing Left Ventricular Hypertrophy in Hypertensive Rats. Chin. J. Hypertens. 16 (01), 33–35.

[B48] Malhi G. S. Mann J. J. (2018). Depression. Lancet 392 (10161), 2299–2312. 10.1016/s0140-6736(18)31948-2 30396512

[B49] Manogaran P. Beeraka N. M. Huang C.-Y. Vijaya Padma V. (2019). Neferine and Isoliensinine Enhance 'intracellular Uptake of Cisplatin' and Induce 'ROS-Mediated Apoptosis' in Colorectal Cancer Cells - A Comparative Study. Food Chem. Toxicol. 132, 110652. 10.1016/j.fct.2019.110652 31255669

[B50] Mazumder A. Cerella C. Diederich M. (2018). Natural Scaffolds in Anticancer Therapy and Precision Medicine. Biotechnol. Adv. 36 (6), 1563–1585. 10.1016/j.biotechadv.2018.04.009 29729870

[B51] Mukherjee P. K. Mukherjee D. Maji A. K. Rai S. Heinrich M. (2009). The Sacred lotus (*Nelumbo nucifera*) - Phytochemical and Therapeutic Profile. J. Pharm. Pharmacol. 61 (4), 407–422. 10.1211/jpp/61.04.0001 19298686

[B52] Nan L. Xuan G. D. (2012). [Influence of Isoliensinine on Activity of CYP3A in Rats]. Zhejiang Da Xue Xue Bao Yi Xue Ban 41 (2), 178–182. CNKI:SUN:ZJYB.0.2012-02-013 22499515

[B53] Newman D. J. Cragg G. M. (2016). Natural Products as Sources of New Drugs from 1981 to 2014. J. Nat. Prod. 79 (3), 629–661. 10.1021/acs.jnatprod.5b01055 26852623

[B54] Paudel K. R. Panth N. (2015). Phytochemical Profile and Biological Activity ofNelumbo Nucifera. Evid. Based Complement. Altern. Med. 2015, 789124. 10.1155/2015/789124 PMC4710907 27057194

[B55] Peng L. S. Jiang X. Y. Li Z. X. Yi T. G. Huang B. Li H. L. (2015). A Simple U-HPLC-MS/MS Method for the Determination of Liensinine and Isoliensinine in Rat Plasma. J. Chromatogr. B 991, 29–33. 10.1016/j.jchromb.2015.03.027 25910234

[B56] Qian J. Q. (2002). Cardiovascular Pharmacological Effects of Bisbenzylisoquinoline Alkaloid Derivatives. Acta Pharmacol. Sin 23 (12), 1086–1092. CNKI:SUN:ZGLL.0.2002-12-0043 12466045

[B93] Richman D. D. (2001). HIV chemotherapy. Nature 410 (6831), 995–1001. 10.1038/35073673 11309630

[B57] Shadel G. S. Horvath T. L. (2015). Mitochondrial ROS Signaling in Organismal Homeostasis. Cell 163 (3), 560–569. 10.1016/j.cell.2015.10.001 26496603 PMC4634671

[B58] Sharma B. R. Gautam L. N. S. Adhikari D. Karki R. (2017). A Comprehensive Review on Chemical Profiling ofNelumbo Nucifera: Potential for Drug Development. Phytother. Res. 31 (1), 3–26. 10.1002/ptr.5732 27667670

[B59] Shen Q. Zuo M. Ma L. Tian Y. Wang L. Jiang H. (2014). Demethylation of Neferine in Human Liver Microsomes and Formation of Quinone Methide Metabolites Mediated by CYP3A4 Accentuates its Cytotoxicity. Chem. Biol. Interact. 224, 89–99. 10.1016/j.cbi.2014.10.014 25451576

[B60] Shen C. Y. Jiang J. G. Yang L. Wang D. W. Zhu W. (2017). Anti-ageing Active Ingredients from Herbs and Nutraceuticals Used in Traditional Chinese Medicine: Pharmacological Mechanisms and Implications for Drug Discovery. Br. J. Pharmacol. 174 (11), 1395–1425. 10.1111/bph.13631 27659301 PMC5429334

[B61] Shu G. Yue L. Zhao W. Xu C. Yang J. Wang S. (2015). Isoliensinine, a Bioactive Alkaloid Derived from Embryos of *Nelumbo nucifera*, Induces Hepatocellular Carcinoma Cell Apoptosis through Suppression of NF-Κb Signaling. J. Agric. Food Chem. 63 (40), 8793–8803. 10.1021/acs.jafc.5b02993 26389520

[B62] Shu G. Zhang L. Jiang S. Cheng Z. Wang G. Huang X. (2016). Isoliensinine Induces Dephosphorylation of NF-Κb P65 Subunit at Ser536 via a PP2A-dependent Mechanism in Hepatocellular Carcinoma Cells: Roles of Impairing PP2A/I2PP2A Interaction. Oncotarget 7 (26), 40285–40296. 10.18632/oncotarget.9603 27244888 PMC5130008

[B63] Sugimoto Y. Nishimura K. Itoh A. Tanahashi T. Nakajima H. Oshiro H. (2015). Serotonergic Mechanisms Are Involved in Antidepressant-like Effects of Bisbenzylisoquinolines Liensinine and its Analogs Isolated from the Embryo of *Nelumbo nucifera* Gaertner Seeds in Mice. J. Pharm. Pharmacol. 67 (12), 1716–1722. 10.1111/jphp.12473 26246025

[B64] Tang G. X. Zhao L. B. Wang X. M. Zhang S. H. Wang J. L. (2007). Protective Effects of Isoliensinine on Acute Lung Injury and Pulmonary Fibrosis Induced by Paraquat. Chin. J. Pharmacol. Toxicol. 21 (06), 462–469. CNKI:SUN:YLBS.0.2007-06-004

[B65] Tian Y. Qian S. Jiang Y. Shen Q. Zheng J. Zhou H. (2013). The Interaction between Human Breast Cancer Resistance Protein (BCRP) and Five Bisbenzylisoquinoline Alkaloids. Int. J. Pharm. 453 (2), 371–379. 10.1016/j.ijpharm.2013.05.053 23742976

[B66] Tomita M. Furukawa H. Yang T.-H. Lin T.-J. (1965). On the Alkaloids of *Nelumbo nucifera* GAERTN. VIII. Studies on the Alkaloids of Loti Embryo. (1). Structure of Isoliensinine, a New Biscoclaurine Type Alkaloid. Chem. Pharm. Bull. 13 (1), 39–43. 10.1248/cpb.13.39 5864280

[B67] Wan D. Han Y. Li F. Mao H. Chen G. (2019). Far Infrared-Assisted Removal of Extraction Solvent for Capillary Electrophoretic Determination of the Bioactive Constituents in Plumula Nelumbinis. Electrophoresis 40 (4), 582–586. 10.1002/elps.201800477 30488648

[B68] Wang X. Liu J. Geng Y. Wang D. Dong H. Zhang T. (2010). Preparative Separation of Alkaloids fromNelumbo nuciferaGaertn by pH-Zone-Refining Counter-current Chromatography. J. Sep. Sci. 33 (4-5), 539–544. 10.1002/jssc.200900561 20063354

[B69] Wang Y. Zhang L. Zhou H. Guo X. Wu S. (2017). K -targeted Strategy for Isolation of Phenolic Alkaloids of *Nelumbo nucifera* Gaertn by Counter-current Chromatography Using Lysine as a pH Regulator. J. Chromatogr. A 1490, 115–125. 10.1016/j.chroma.2017.02.022 28236459

[B70] Wu S. Sun C. Cao X. Zhou H. Zhang H. Pan Y. (2004). Preparative Counter-current Chromatography Isolation of Liensinine and its Analogues from Embryo of the Seed of *Nelumbo nucifera* GAERTN. Using Upright Coil Planet Centrifuge with Four Multilayer Coils Connected in Series. J. Chromatogr. A 1041 (1-2), 153–162. 10.1016/j.chroma.2004.05.003 15281264

[B71] Xiao J.-H. Zhang J.-H. Chen H.-L. Feng X.-L. Wang J.-L. (2005a). Inhibitory Effects of Isoliensinine on Bleomycin-Induced Pulmonary Fibrosis in Mice. Planta Med. 71 (3), 225–230. 10.1055/s-2005-837821 15770542

[B72] Xiao J. H. Zhang Y. L. Ding L. L. Feng X. L. Wang J. L. (2005b). Effects of Isoliensinine on Proliferation of Porcine Coronary Arterial Smooth Muscle Cells Induced by Phenylephrine. Yao Xue Xue Bao 40 (2), 105–110. 15875663

[B73] Xiao J. H. Zhang Y. L. Feng X. L. Wang J. L. Qian J. Q. (2006). Effects of Isoliensinine on Angiotensin II-Induced Proliferation of Porcine Coronary Arterial Smooth Muscle Cells. J. Asian Nat. Prod. Res. 8 (3), 209–216. 10.1080/1028602042000325609 16864426

[B74] Xie Y. Zhang Y. Zhang L. T. Zeng S. X. Guo Z. B. Zheng B. D. (2013). Protective Effects of Alkaloid Compounds from Nelumbinis Plumula on Tert-Butyl Hydroperoxide-Induced Oxidative Stress. Molecules 18 (9), 10285–10300. 10.3390/molecules180910285 24064445 PMC6269732

[B75] Xu J. Y. Peng G. L. Wang J. L. (2005). Protective Effect of Isoliensinine on Experim Ental Cerebral Ischemia Injury. Chin. Hosp. Pharm. 25 (11), 1026–1029. CNKI:SUN:ZGYZ.0.2005-11-012

[B76] Yang X. Huang M. Yang J. Wang J. Zheng S. Ma X. (2017). Activity of Isoliensinine in Improving the Symptoms of Type 2 Diabetic Mice via Activation of AMP-Activated Kinase and Regulation of PPARγ. J. Agric. Food Chem. 65 (33), 7168–7178. 10.1021/acs.jafc.7b01964 28745497

[B77] Yang G.-M. Sun J. Pan Y. Zhang J.-L. Xiao M. Zhu M.-S. (2018). Isolation and Identification of a Tribenzylisoquinoline Alkaloid from *Nelumbo nucifera* Gaertn, a Novel Potential Smooth Muscle Relaxant. Fitoterapia 124, 58–65. 10.1016/j.fitote.2017.10.020 29108933

[B78] Yildiz M. Oktay A. A. Stewart M. H. Milani R. V. Ventura H. O. Lavie C. J. (2020). Left Ventricular Hypertrophy and Hypertension. Prog. Cardiovasc. Dis. 63 (1), 10–21. 10.1016/j.pcad.2019.11.009 31759953

[B79] Yu L. Shen Q. Zhou Q. Jiang H. Bi H. Huang M. (2013). *In vitro* characterization of ABC Transporters Involved in the Absorption and Distribution of Liensinine and its Analogs. J. Ethnopharmacol. 150 (2), 485–491. 10.1016/j.jep.2013.08.061 24036064

[B80] Yu W. G. Cheng T. X. Zhang H. W. Xie P. Zhang Y. X. Zhang D. M. (2007). Effect of Liensinine on C-Fos Protein Expression in Ratswith FocalCerebral Ischemia. Lishizhen Med. Materia Medica Res. 18 (03), 536–537. CNKI:SUN:SZGY.0.2007-03-013

[B81] Zang Y. L. Yang G. M. Li P. Pan Y. (2015). Protective Effects of Four Alkaloids of Embryo Loti on H_2_O_2_-Induced Oxidative Damage of Vascular Endothelial Cells. Chin. J. Biochem. Pharm. 35 (03), 1–5. CNKI:SUN:SHYW.0.2015-03-001

[B82] Zhang F. Wang J. L. Qiang J. Q. (2000a). Effects of Isoliensinine on Experimental Arrhythmia and Myocardium Action Potential of Guinea Pig. Chin. Traditional Herbal Drugs 31 (10), 35–37. CNKI:SUN:YLBS.0.2000-04-011

[B83] Zhang F. Wang J. L. Qiang J. Q. (2000b). Effects of Isoliensinine on Hemodynamics and Smooth Muscle Contraction in Anesthetized Rats. Chin. J. Pharmacol. Toxicol. 14 (4), 296–299. CNKI:SUN:ZCYO.0.2000-10-020

[B84] Zhang X. Wang X. Wu T. Li B. Liu T. Wang R. (2015). Isoliensinine Induces Apoptosis in Triple-Negative Human Breast Cancer Cells through ROS Generation and P38 MAPK/JNK Activation. Sci. Rep. 5, 12579. 10.1038/srep12579 26219228 PMC4518223

[B85] Zhang Z. X. (2010). Anti-dementia Effects of Isoliensinine, Liensinine and Neferine Master's Degree. Hubei, China: Wuhan Huazhong University of Science and Technology.

[B86] Zhao X. Shen J. Chang K. J. Kim S. H. (2014). Comparative Analysis of Antioxidant Activity and Functional Components of the Ethanol Extract of lotus (*Nelumbo nucifera*) from Various Growing Regions. J. Agric. Food Chem. 62 (26), 6227–6235. 10.1021/jf501644t 24932940

[B87] Zheng H.-C. (2017). The Molecular Mechanisms of Chemoresistance in Cancers. Oncotarget 8 (35), 59950–59964. 10.18632/oncotarget.19048 28938696 PMC5601792

[B88] Zhou H. Jiang H. Yao T. Zeng S. (2007). Fragmentation Study on the Phenolic Alkaloid Neferine and its Analogues with Anti-HIV Activities by Electrospray Ionization Tandem Mass Spectrometry with Hydrogen/deuterium Exchange and its Application for Rapid Identification Ofin Vitro Microsomal Metabolites of Neferine. Rapid Commun. Mass. Spectrom. 21 (13), 2120–2128. 10.1002/rcm.3070 17546644

[B89] Zhou H. Li L. Jiang H. Zeng S. (2012). Identification of Three New N-Demethylated and O-Demethyled Bisbenzylisoquinoline Alkaloid Metabolites of Isoliensinine from Dog Hepatic Microsomes. Molecules 17 (10), 11712–11720. 10.3390/molecules171011712 23027371 PMC6268788

[B90] Ziegler J. Facchini P. J. (2008). Alkaloid Biosynthesis: Metabolism and Trafficking. Annu. Rev. Plant Biol. 59, 735–769. 10.1146/annurev.arplant.59.032607.092730 18251710

