# Supplementary material for: Isoliensinine: A Natural Compound with “Drug-Like” Potential
Source: Front Pharmacol. 2021 Apr 22;12:630385. doi: 10.3389/fphar.2021.630385 (PMC8100667; doi:10.3389/fphar.2021.630385)
Supplement: Supplementary file 1 [file table1.docx]

TABLE 1 **|** Pharmacological activity of isoliensinine

| **Pharmacological activity** | **Model** | **Action and Mechanism** | **Ref.** |
| --- | --- | --- | --- |
| Anti-cancer activity | Multiple cancers (HeLa, A549, MCF-7, PC-3, Hep3B, H1299, LO2); Apoptosis- resistant cells (caspase-3/-7/-8 deficient MEFs and Bax/Bak double knockout MEFs) | Enhancers of autophagy  LC3-I↑, LC3-II↑, p-AMPK↑, TSC2↑, p-P70S6K↓ | (Law, Chan et al. 2014) |
|  | Triple-negative human breast cancer cells (MCF-10A, MDA-MB-436, MDA-MB- 468, MDA-MB-231, MCF-7) | P21↑, CyclinE↓, Bcl-2/Bax↓, cleaved caspase-3↑, cleaved PARP-1↑, ROS↑, p-P38↑, p-JNK↑ | (Zhang, Wang et al. 2015) |
|  | Hepatocellular Carcinoma Cells (HepG2, Huh-7, H22); Xenografts nude mice transplanted Huh-7; Kunming mice of transplanted H22 | Bcl-2↓, Bcl-xL↓, MMP9↓, activity of caspase-3↑, activity of NF-kB↓, p-P65↓, interaction of PP2A/ I2PP2A↓ | (Shu, Yue et al. 2015, Shu, Zhang et al. 2016) |
|  | Hepatocellular Carcinoma Cell (HepG2, Huh-7, H22, HL-7702, Hepa1-6, Hep3B);  Huh-7 xenografts Nude Mice | Chemosensitized by dauricine  miR-199a↑, HK2 ↓, PKM2↓ | (Li, Qiu et al. 2018) |
|  | Colorectal cancer cells (HCT-15) | Chemosensitizing activity on cisplatin, ROS↑, MMP↓,Bcl-2↓, cleaved caspase-3↑, cleaved PARP↑, Cyto C↑, p-P38↑, P38↑, PI3K↓, p-AKT↓ | (Manogaran, Beeraka et al. 2019) |
| Cardiovascular protective activity | Porcine coronary arterial smooth muscle cells | Inhibition of proliferation of porcine coronary arterial smooth muscle cells induced by angiotensin II  PDGF-β↓，bFGF↓, c-fos↓, c-myc ↓，hsp70↓ | (Xiao, Zhang et al. 2005, Xiao, Zhang et al. 2006) |
|  | Isolated mesenteric vascular smooth muscle | Relaxing abnormal smooth muscle contractions | (Yang, Sun et al. 2018) |
|  | Aortic tissues of male spontaneously hypertensive rats; Vascular smooth muscle cell (VSMCs) | Anti-hypertension; Ameliorating aortic remodeling collagen I↓, α-SMA↑, p-MYPT1↓, ROCK↓, RhoA transposition↓ | (Li, Wo et al. 2019) |
|  | Isolated rabbit left ventricular myocytes | Antiarrhythmic  I _NaL_↓, I _CaL_↓  EADs↓, DADs↓ | (Liu, Hu et al. 2020) |
| Antioxidant activity | Human hepatocellular HepG2 | Anti-oxidative stress  ROS↓, TBARS↓, LDH ↓  GSH↑ | (Xie, Zhang et al. 2013) |
|  | D-Galactose-induced aged mice | Anti-aging, MDA↓,  SOD↑, GSH-Px↑ | (Liu SL, Hao YR et al. 2011, Shen, Jiang et al. 2017) |
| Anti-virus activity | H9 | Anti-HIV | (Kashiwada, Aoshima et al. 2005, Zhou, Jiang et al. 2007) |
| Anti-depressant activity | Mice (the forced swimming test) | Anti-immobility effects  5-HT _1A_ receptor | (Sugimoto, Nishimura et al. 2015) |
| Improving Alzheimer’s disease activity | 96-well plate contained BChE | Inhibition of the activity of BChE | (Lin, Wang et al. 2013) |
| Anti-diabetic activity | Rat skeletal muscle cells, L6;  the KK-Ay rat | GLUT4↑, p-AMPK↑, p-ACC↑, PPARγ↓, SREBP-1c↓, ACC↓ | (Yang, Huang et al. 2017) |
| Anti-pulmonary fibrosis | Male Kunming mice | MDA↓, hydroxyproline↓，ALP↓  SOD↑, TGF-β1↓，TNF-α↓,MMP2↓ | (Xiao, Zhang et al. 2005) |
