# Supplementary material for: Isoliensinine: A Natural Compound with “Drug-Like” Potential
Source: Front Pharmacol. 2021 Apr 22;12:630385. doi: 10.3389/fphar.2021.630385 (PMC8100667; doi:10.3389/fphar.2021.630385)
Supplement: Supplementary file 2 [file datasheet1.docx]

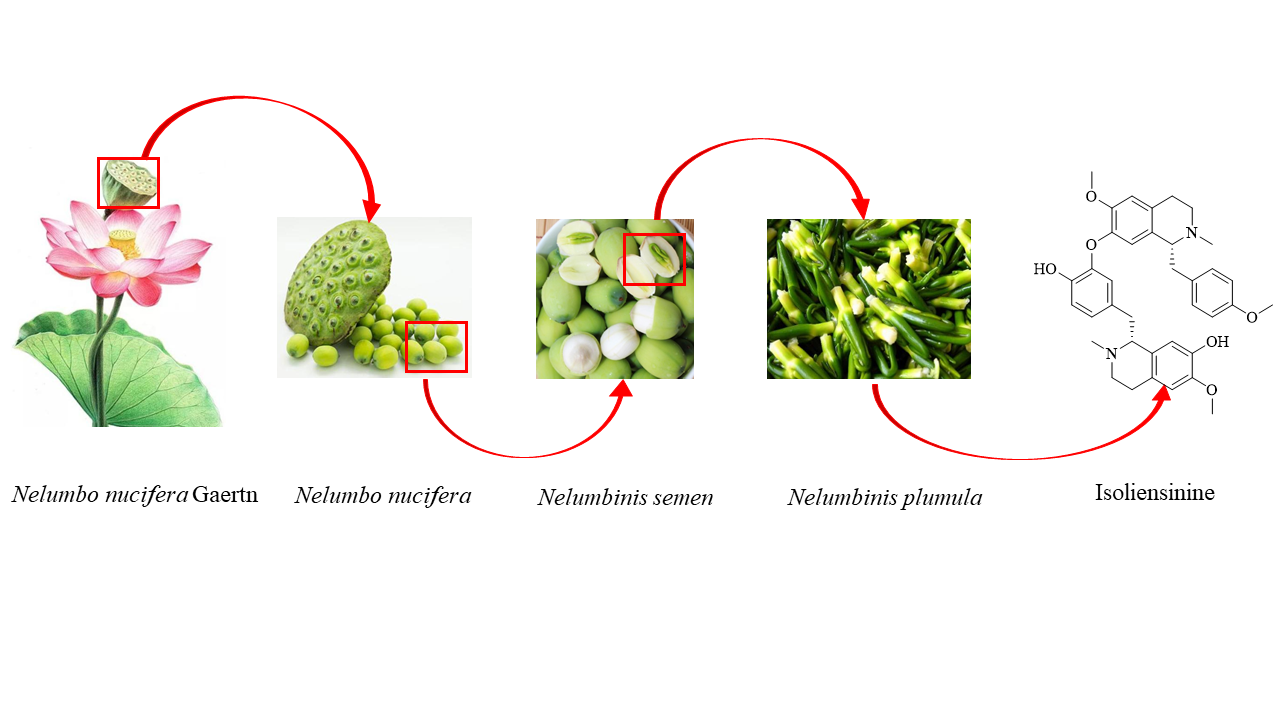


Figure1. The source of isoliensinine

Figure 2(A).The chemosynthesis of isoliensinine

Figure 2(B). The biosynthesis of Bisbenzylisoquinoline alkaloids


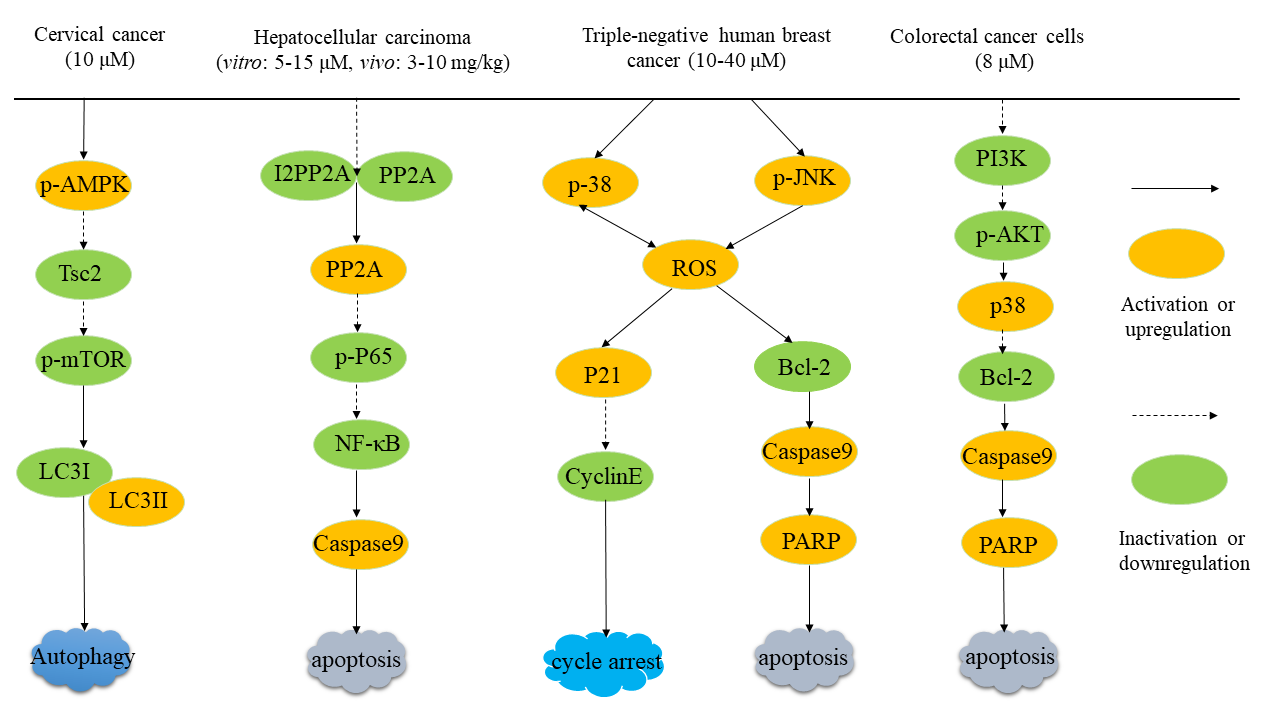


Figure3. The anti-cancer activity and dosage of isoliensinine in various tumors
